# Applying Bayesian checks of cancellation axioms for interval scaling in limited samples

**DOI:** 10.3758/s13428-025-02844-7

**Published:** 2025-10-07

**Authors:** Sanford R. Student, Wyatt S. Read

**Affiliations:** University of Delaware School of Education, 113 Willard Hall Education Building, 16 West Main Street, Newark, DE 19716 USA

**Keywords:** Item Response Theory, Rasch model, Additive conjoint measurement, Interval scale, Latent variable modeling

## Abstract

Interval scales are frequently assumed in educational and psychological research involving latent variables, but are rarely verified. This paper outlines methods for investigating the interval scale assumption when fitting the Rasch model to item response data. We study a Bayesian method for evaluating an item response dataset’s adherence to the cancellation axioms of additive conjoint measurement under the Rasch model, and compare the extent to which the axiom of double cancellation holds in the data at sample sizes of 250 and 1000 with varying test lengths, difficulty spreads, and levels of adherence to the Rasch model in the data-generating process. Because the statistic produced by the procedure is not directly interpretable as an indicator of whether an interval scale can be established, we develop and evaluate procedures for bootstrapping a null distribution of violation rates against which to compare results. At a sample size of 250, the method under investigation is not well powered to detect the violations of interval scaling that we simulate, but the procedure works quite consistently at *N* = 1000. That is, at moderate but achievable sample sizes, empirical tests for interval scaling are indeed possible.

## Introduction

Most psychometric models and most quantitative analyses of scores based upon them assume an interval interpretation of person and item parameters, but this foundational assumption is rarely tested in practice (Mislevy, 2018). Yet, building on foundational contributions from Karabatsos (2001), Domingue (2014) provides a method for testing the plausibility of an interval interpretation for parameters of the Rasch (1960) model fit to a given dataset – what we term Bayesian Conjoint Checks, or BCC. This paper examines the method at commonly encountered sample sizes and proposes an approach for interpreting the results in relation to a null distribution, thereby making BCC more accessible and interpretable for researchers in the behavioral sciences. We demonstrate that investigation and falsification of the interval properties of latent variables is indeed possible, albeit not at all sample sizes.

Domingue (2014)’s approach builds on the foundational work of Karabatsos (2001), who developed methods to investigate the possibility of constructing a scale with equal-interval properties (Stevens, 1946) – both by improving the alignment of the method with its mathematical basis and by providing a free, easy-to-use R package that makes the methods accessible to any researcher. This line of methodological research represents a major advancement for the prospect of empirically establishing the existence of an interval scale in psychological measurement (Karabatsos, 2018), but the statistic produced by the method requires additional context for interpretation because it may take on very different values as a function of sample size, test length, and true item parameters. Given this, we aim to understand how the method performs at relatively small sample sizes, and to evaluate a proposed bootstrapping approach to improve the interpretability of the statistic produced by this method. This study aims to build an understanding of how BCC can be applied in practice, as well as the circumstances under which it can reliably discriminate data that can be used to construct an interval scale from data that cannot.

For nearly as long as it has existed, the Rasch model (Rasch, 1960) has been framed as an instantiation of additive conjoint measurement (ACM; Krantz et al., 1971; Luce & Tukey, 1964), an axiomatic framework for establishing the interval properties of a scale on which are measured three variables: a dependent variable, and two independent variables of which the dependent variable is a non-interacting function (see e.g., Brogden, 1977; Keats, 1967; Perline et al., 1979). Karabatsos (2001) contributed what we term BCC: a Markov Chain Monte Carlo method for estimating the proportion of violations of single and double cancellation, axioms of ACM that are necessary for interval scaling, in a dataset of dichotomous responses to test items. Domingue (2014) then improved upon this methodology and provided the *ConjointChecks* R package to make its implementation accessible (Domingue, 2012a). However, the performance and application of BCC using Domingue’s implementation have only been investigated in the original 2014 paper and, to a greater degree, in the dissertation that preceded it (Domingue, 2012b). All of the simulations investigating the BCC method were based on datasets of 15,000 sets of responses to 45 items. This is certainly an important starting point, as the interval properties of score scales (or lack thereof) can be particularly consequential in the sorts of high-stakes educational testing settings (Ballou, 2009; Briggs, 2013; Briggs & Domingue, 2013) where such large samples are common (Student, 2024). However, researchers conducting educational and psychological research often analyze test and survey data using parametric statistics that implicitly rely on variables having interval scale properties (such as tests of mean differences and multiple regression). Sample sizes in these studies may not be as large as those used in Domingue (2012b, 2014)’s simulations, and in many cases will be *far* smaller. Even in research explicitly focusing on ACM in educational and psychological measurement, this is not infrequently the case – for example, in a study of the adherence of item responses to a college general knowledge test administered to 990 undergraduates (Green, 1986), or a set of items making up a tool for likelihood of parole violations administered to 490 individuals released after conviction of a crime (Perline et al., 1979). There is little reason why interval scaling should be any less of a concern at these sample sizes if the aim of those studies is to investigate the adherence of item response data to the axioms of ACM. Thus, this study contributes new insight into whether, and how, BCC can be applied at commonly encountered sample sizes. Notably, although BCC provides an important quantity of interest – the proportion of violations of single and double cancellation – the method itself does not yield any insight into the extent to which that quantity may be problematic or not. That is, one cannot know from the result alone whether the data under analysis are compatible with interval scaling based on ACM or not. This paper investigates how the statistic produced by BCC is distributed at moderate sample sizes, and investigates the extent to which bootstrapped null distributions can be generated to potentially create a meaningful basis for interpreting the result of applying the procedure to real data.

### Aims and research questions

Our overarching motivating question is: *can we apply BCC at moderate sample sizes, and if so, how?* To answer this question, we pursue two closely related sequential aims. The first is to investigate the behavior of Domingue (2014)’s Bayesian check of single and double cancellation, BCC, to assess the extent to which the statistic produced by the procedure appears to differ systematically in its value according to sample size, test length, and extent of unequal item discrimination. The second is to develop a method to make the results of this procedure more interpretable by using simulation to generate a null distribution against which to compare the statistic produced by BCC.

We thus address the following research questions:

1. At moderate sample sizes, to what extent do deviations from the Rasch model and differences in test length/item difficulty produce violations of Domingue (2014)’s Bayesian checks of single and double cancellation under ACM?
2. When violations are present, to what extent does generating simulated null distributions help to distinguish between data that are compatible vs. incompatible with interval scaling?

### Background

Below, we provide a brief overview of the issues at hand. These are not comprehensive reviews, as this study primarily focuses on an enhanced approach to applying an existing method. Instead, we focus on the aspects of the prior literature that directly inform the present study.

### Metric properties of scales in educational and psychological measurement

The metric properties of scales in educational and psychological measurement have been of interest for nearly the entire history of the field (Briggs, 2021; Thurstone, 1927). In particular, S.S. Stevens’ distinction between *ordinal* scales and *interval* scales (Stevens, 1946), and their associated permissible operations, has received a great deal of attention due to its implications for nearly all parametric data analysis methods commonly used in the social sciences. This is largely a matter of how one can make sense of differences along the scale. Briefly, locations on an ordinal scale can be ordered, but the differences between them are not comparable – a commonly cited instance of ordinal measurement is the Mohs hardness scale, on which numbers from 1 to 10 correspond solely to which minerals a given substance can and cannot scratch (Tabor, 1954) but do not represent consistent amounts of hardness in any physical sense. In the present context, the concern for psychological research is that many studies use sum (number-correct) scores as predictors and outcome variables. As measures of the underlying construct of interest, such scores support only an ordinal interpretation that is sometimes quite useful and sometimes a serious statistical limitation for subsequent analysis (Domingue et al., 2022; McNeish & Wolf, 2020; Sijtsma et al., 2024). In contrast, *interval* scales produce meaningful^1^ differences, such that the amount of change represented by, e.g., 4 units is the same no matter where the change occurred – the Celsius and Fahrenheit temperature scales are examples of interval scales. The promise of Rasch modeling and ACM is that person scores, as well as item difficulty parameters, based on the model can take on an interval interpretation if the data fit the model sufficiently (Keats, 1967; Kyngdon, 2011; Luce & Tukey, 1964; Perline et al., 1979). *Ratio* scales are distinguished from interval scales by having a “true zero” such that zero represents the absence of the property being measured – physical scales, including measures of length, temperature in Kelvin, and so on are ratio scales. However, the key distinction is between ordinal and interval scales because so much data analysis in educational and psychological science involves the use of distances between observations. It is beyond the scope of this manuscript to provide an overview of all of the situations in which the interval properties of a scale are relevant to analytic techniques because the issue is so foundational, but an extensive literature deals with both the theoretical and empirical consequences of using ordinally scaled measures as if they were interval-scaled without evidence for doing so (see, e.g., Ballou, 2009; Bond & Lang, 2013; Briggs, 2013; Briggs & Domingue, 2013; Domingue et al., 2022; Michell, 1986, 1997, 2008). In short, because differences on an ordinal scale are not truly comparable, nearly any statistic beyond the quantiles of a distribution may be distorted when ordinal-scaled data are used as if they are interval-scaled.

### The Rasch model and additive conjoint measurement: A brief review

The metric properties of scales are of particular interest when conducting statistical analysis of results on a survey or test instrument intended to measure a construct that is not directly observable but is hypothesized to drive responses to the questions that make up the instrument, i.e., a latent variable (Bollen, 2002; Maul, 2013; Maul et al., 2016). The fundamental issue is that the metric properties of the scores are not known and generally cannot be directly investigated (Domingue et al., 2022). However, among the many latent variable models for scoring these instruments, the Rasch model (Rasch, 1960) has received particular attention as a potential route to producing interval-scaled scores (Briggs, 2013, 2019; Domingue, 2014; Karabatsos, 2001, 2018; Perline et al., 1979). This is because when item response data fit it, the Rasch model can arguably be viewed as an example of ACM (Luce & Tukey, 1964), unlike more flexible models in the item response theory paradigm (Birnbaum, 1968; Lord, 1980). ACM is a set of mathematical axioms describing relationships between two independent variables and a third variable depending upon them that, when they hold, are sufficient to verify that the three variables can be measured on a common, interval scale (Krantz et al., 1971). For the Rasch model, the three variables are found in the item response function:

1 $$\text{ln}\left(\frac{P\left({X}_{ij}=1\right|{\theta }_{i},{b}_{j})}{1-P\left({X}_{ij}=1\right|{\theta }_{i},{b}_{j})}\right)={\theta }_{i}-{b}_{j}$$

In this model, ${\theta }_{i}$ is a measure of examinee standing on the latent variable of interest (often called “ability”); ${b}_{j}$ is a measure of an item’s standing on that same latent variable (often called difficulty); ${X}_{ij}$ is examinee *i*’s response to item *j*, taking on values of either 0 (i.e., incorrect, disagree, etc.) or 1 (i.e., correct, agree, endorse, etc.). $\text{ln}\left(\frac{P\left({X}_{ij}=1\right|{\theta }_{i},{b}_{j})}{1-P\left({X}_{ij}=1\right|{\theta }_{i},{b}_{j})}\right)$ is the log-odds, or logit, of ${X}_{ij}$ taking on value 1, and under the Rasch model, this is defined by an additive relation between person ability and item difficulty. In theory, the axioms of ACM can be applied to these three values – item difficulty and person ability as the independent variables, and the logit of a correct response as the dependent variable – because of the additive relation, ${\theta }_{i}-{b}_{j}$, that produces the dependent variable from the independent ones. In practice, due to the sufficiency of observable values to estimate these parameters, these parameters are not typically tested directly for adherence to ACM, a point to which we return below (Domingue, 2014; Perline et al., 1979).

### Testing for single and double cancellation

Though not sufficient on their own to *prove* that variables follow the axioms of ACM, the axioms of single and double cancellation have received the most attention in the Rasch-ACM literature because unlike other axioms of ACM^2^ they are feasibly testable using psychological data (Michell, 1990). The earliest attempt to apply ACM to the Rasch model was Perline et al. (1979). In that study, single and double cancellations were investigated with empirical *p* values for responses from 490 individuals released on parole. The authors concluded based on their analysis that the data did not satisfy the axioms of ACM, but they did not account for sample size or sampling variability in any way (Karabatsos, 2001). Karabatsos (2001) partially reanalyzed Perline et al.’s data in the context of his introduction of Bayesian methods for assessing violations of the axioms of ACM, laying the foundation for the present study. Karabatsos’ approach (a) was framed in terms of nonparametric item response theory, and therefore may have limited appeal in a field widely reliant on parametric models to define score scales, and (b) was more restrictive than ACM actually requires, as described by Domingue (2014, p. 8). Kyngdon (2011) explicitly connects Karabatsos’ approach to the Rasch model, potentially opening the door to Bayesian checks of adherence to ACM for this widely popular measurement model. Complementing this work, Domingue (2014) improved upon Karabatsos’ approach methodologically, bringing the implementation in line with the requirements of single and double cancellation under ACM. A heuristic description of this approach – what we term BCC – is provided next.

Under the Rasch model, the proportion of correct responses across all of the items is a sufficient statistic for estimating a person’s $\theta$; the proportion of correct responses to a given item across all of the examinees is a sufficient statistic for estimating the item’s difficulty. Given this, Domingue (2014) focuses on evaluating the double cancellation axioms (and, as a byproduct, single cancellation as well) of ACM in terms of individuals’ sum/proportion-correct scores, items’ number/proportion correct, and the empirical proportion of individuals with each possible sum score correctly answering each item. Because these independent variables are sufficient statistics for estimating the parameters of the Rasch model, they order examinees and items identically to how they would be ordered by the Rasch model’s estimated parameters, and Domingue’s test can provide insight into whether a given dataset is compatible with a hypothesis of an interval scale *before the scale is constructed* (see the original paper for mathematical details). The Rasch model is then used to transform these sufficient statistics to their interval scale. That is, *if* Domingue’s checks of double cancellation provide evidence that the dataset is compatible with a hypothesis of interval scaling, *then* the Rasch model can be fit to the data, and the resulting person and item parameters can be used as interval-scaled measures. This method is what we refer to as BCC.

The statistic provided by BCC is an estimate of the proportion of checks of double cancellation that violate the axiom. For example, the procedure might indicate that, say, 8.52% of the comparisons made in the procedure indicated a violation. This statistic does not have an associated hypothesis test. As such, additional information is needed to determine if the proportion of violations is acceptable – that is, if the proportion of violations is in line with what one would expect from data generated from the Rasch model, which are inherently interval-scalable. Domingue (2014) makes this judgment by comparing an estimate for real data to the mean proportion of violations in datasets generated from the Rasch model with similar distributions of $\theta$ and a similar number of items – effectively, a null expected value for the proportion of violations. For this approach to be viable, it is important to understand the properties of that null expected value in terms of what aspects of the data-generating mechanism drive increasing proportions of violations. As the sample sizes get smaller, this expected value also becomes more unstable, motivating our pursuit of a null *distribution* rather than a single value for comparison. We return to these points in describing our simulation and real data analysis approach; for now, we focus on what a “violation” represents.

Imagine a table in which rows represent groups of individuals with a given sum score; columns represent items, ordered by the proportion of correct responses (empirical *p* values); and cells contain the proportion of individuals with that score answering that item correctly. The essential logic of BCC relies on repeated draws of “3-matrices”, tables subsetted from this full data table to compare the empirical *p* values of correct responses to three items from individuals with three sum scores. For example, Table 1 illustrates a potential 3-matrix from a hypothetical 20-item test.

**Table 1:** Example three-matrix for testing double cancellation

	Item *X*	Item *Y*	Item *Z*
Sum score 6	0.22	0.61	0.70
Sum score 9	0.36	0.72	0.76
Sum score 14	0.45	0.69	0.81

Let $p(s, i)$ represent the empirical *p* value for sum score group *s* and item *i.* Then, relations involving $p(9, Y)$ do not appear to follow the axioms of single or double cancellation; the former would imply that $p(9, Y) < p(14, Y)$, while the latter would imply that $p(6, Z) > p(9, Y)$ given the relations $p\left(6, Y\right)>p(9, X)$ and $p\left(9, Z\right)>p(14, Y)$. If these values had been measured without sampling variability, we would reject the claim that the relations between these variables support an interval scale, because single and double cancellation would be violated. However, they *are* measured with error, as noted above (Domingue, 2014; Green, 1986). This introduces uncertainty into every relation in this 3-matrix, whether or not the underlying relations adhere to single and double cancellation. Based on the values alone, we no longer know. We point out that this issue is especially acute at smaller sample sizes, where sampling variance is largest.

Given this, Domingue (2014) applies an estimation approach based on Markov chain Monte Carlo (MCMC), the Metropolis-Hastings algorithm, and Bayesian credible intervals to attempt to disentangle (1) violations of double cancellation that could plausibly result from sampling variability, from (2) violations unlikely to have arisen by chance from a data-generating process compatible with ACM. The technical details of BCC involving Markov chain Monte Carlo jumping distributions are provided in the appendix of the original study; the result of the analysis is that a 95% credible interval based on the requirements of double cancellation (and trivially, single cancellation) is constructed for each cell under analysis, and the cell is deemed to have violated the axioms if its empirical *p* value falls outside the credible interval.

Domingue (2014) investigated two additional issues: how to select 3-matrices, and how to summarize violations. For the former issue, he considered two possibilities: evaluating all 3-matrices for adjacent sets of items and sum scores, or randomly selecting a large number of 3-matrices among all possible choices^3^. He reported that the latter choice, using 5000 random 3-matrices is more stringent than the former, and that is the approach we use in this study. For the latter issue, Domingue considered either an unweighted mean proportion of violations across items or a weighted version where violations are weighted by the number of individuals with the associated sum score to reduce outlier-based volatility. The latter approach led to lower proportions of violations, but more contrast between item responses compatible with ACM and those violating the axioms. For this reason, we focus on the weighted approach below.

### Sample size and the assessment of interval scaling

As noted above, Domingue (2014) presents analyses of simulated and real data with many thousands of sets of responses, given the motivation of the study in terms of sample sizes encountered in state educational testing (Domingue, 2012b), but such samples are uncommon in many research settings. When samples are smaller, the sampling variability of statistics of interest, such as empirical item *p* values, inevitably increases. It is widely recognized that as a result, when sample sizes are small enough (e.g., below 500 based on common rules of thumb), it is generally inadvisable to estimate the parameters of almost any item response model (for contemporary and foundational examples of this guidance, see, e.g., Bandalos, 2018; Lord, 1983). The Rasch model, with its simple one-parameter functional form, has been framed as an exception to this best practice, with seminal work on Rasch modeling using a sample of fewer than 50 students (Wright & Stone, 1979). Yet, this practice has been called into question based on the instability of the results (Chen et al., 2014), and fitting a Rasch model to learn useful information about one’s test items (assuming that this can be done in small samples at all) is not necessarily the same as producing a scale with interval properties, or even producing a scale that meaningfully captures generalizable item response patterns (see p. 1043-1045 and Supplemental Figure S8 of Domingue et al., 2024). Given that noise from sampling variability affects the estimation of the parameters of even a simple one-parameter logistic model (i.e., the Rasch model), one cannot assume that BCC functions as intended at the sample sizes considered in other studies of ACM and the Rasch model (Green, 1986; Perline et al., 1979), and that is why we focus on smaller samples in this study. We take it as a given in this study that the metric properties of scales in educational and psychological research should be of interest to researchers using those scales. It is therefore important to understand if, at smaller sample sizes, the proportion of violations produced by BCC does, in fact, change with aspects of the data-generating process such as departures from the Rasch model, variability in item difficulty, and length of the test (this is the subject of our first simulation study).

What we find peculiar about the relative lack of attention given to interval scaling in applied educational and psychological research is that nominally, the procedures for constructing a scale with interval properties are already relatively accessible. As noted above, it is frequently claimed that the Rasch model can be estimated and used at moderate sample sizes (Linacre, 2005; Wright & Stone, 1979), and software for Rasch analysis is freely and widely available (e.g., Chalmers, 2012; Mair and Hatzinger, 2007; Rizopoulos, 2006). Assuming that one accepts that the model can be framed as ACM^4^, Domingue’s *ConjointChecks* R software package (Domingue, 2012a) can be used to implement BCC in only a few lines of code. If this is nominally all that stands between, on the one hand, the use of a sum score with questionable metric properties (McNeish, 2023; McNeish & Wolf, 2020) in a model, and on the other, a Rasch model-based estimate with potentially interval scale properties, why do so few researchers bother to try to fit a Rasch model at all, much less use BCC to support claims of an interval scale (Mislevy, 2018)? A cynic might suggest that most researchers are either unaware of or uninterested in the distinction between ordinal and interval scales (Briggs & Betebenner, 2009), but we speculate that the issue is not solely one of disinterest: researchers who do consider these issues important still may not know what to do with the results of running *ConjointChecks* on their data because the proportion of violations detected by the procedure is only interpretable relative to a criterion based on data generated from the Rasch model (Domingue, 2012b, 2014). This may be evident in the fact that although Domingue (2014) has been cited 67 times per Google Scholar as of this writing, a review of the citing literature found just a handful of applications of the methods developed in that paper – most of the citations are either references to Domingue’s analysis of reading assessment data or general allusions to the existence of the methods he developed. Two applications we did find illustrate different ways of using the method – neither of which, we contend, fully capitalizes on its potential. In one example (Domingue & Dimitrov, 2021), two different scoring methods are analyzed using BCC, and the results are compared to make an *ordinal* claim about which method produces scores that are *more* compatible with an interval interpretation. That is, no claim is made about either method producing interval-scaled scores or not – just that one seems to come closer than the other. Though valuable for its goals, this type of analysis does not support the direct assessment of whether one can claim that a given scale has interval properties. Second, an analysis of the measurement of effort (Steele, 2023) uses BCC just to validate the use of the Rasch model in general via simulation. However, that analysis assumes that, because BCC is based on 95% credible intervals, violation rates above 0.05 automatically indicate a violation of interval scaling (referred to in Steele as quantitative structure). This strikes us as an unwarranted conclusion, because all of Steele’s data were simulated from the Rasch model – that is, the underlying data-generating mechanism does, by definition, follow an interval scale. So if empirical violation rates are above 0.05 at smaller samples or larger test lengths, as Steele finds, that does not, to us, indicate that those datasets are incompatible with an interval interpretation. It signals that BCC does not always support the use of a 5% cutoff, and that the sensitivity of violation rates to data-generating conditions warrants further investigation. Below, we take this up via our first simulation study.

## Detecting violations of single and double cancellation at smaller sample sizes: Simulation Study 1

Our first Monte Carlo simulation study is about the sensitivity of BCC to different aspects of the data-generating process for item responses. As noted above, we do not think that it is warranted to automatically assume a cutoff of 5% for evidence of non-interval scaling, even though BCC is based on 95% credible intervals, due to existing evidence that data generated *from the Rasch model* can regularly produce violation rates in excess of 5%. Our aim, therefore, is to determine the extent to which BCC produces systematically higher or lower proportions of violations as a function of sample size, test length, difficulty spread, and item discrimination spread (including unit discrimination, i.e., Rasch data generation). This tells us if further work to develop null distributions for the proportion of violations is likely to yield meaningful results.

### Study design

The study is based upon fully crossing the following design variables:

1. Sample size: 250 or 1000 examinees

These sample sizes were chosen because they are large enough to sufficiently power common designs in educational and psychological research, such as tests of mean differences with moderate effect sizes or multiple regression with several covariates, but are also distinct enough from Domingue’s original sample size of 15,000 to warrant investigation. They are also similar to sample sizes of prior studies in this area (Green, 1986; Perline et al., 1979).

2) Test length: 20 or 50 items

These test lengths were chosen because they represent typical lengths of instruments used in educational and psychological research. A 20-item test of dichotomous items will typically produce acceptable reliability: an estimate of coefficient $\alpha$ (Cronbach, 1951) around 0.8, which is typically deemed “good enough” for use in research (Bandalos, 2018). A 50-item test made up of dichotomous items is more similar to a scenario in which researchers are using an existing, highly reliable instrument such as a state academic achievement test or commercially available assessment.

3) Item difficulty distribution: $N(\mu = 0,{\sigma }^{2}= 0.25)$ or $N(0, 1.44)$

Item difficulty is drawn from a normal distribution based on the researchers’ experience with real-world educational testing data; although it is not necessarily a best practice to create test forms with normally distributed item difficulties, in our experience with educational testing this is more common than uniformly distributed difficulty.

4) Data-generating item discrimination: Rasch (unit discrimination), $unif\sim (0.8, 1.2)$, $unif(0.5, 1.7)$

We generate data from the Rasch model as a baseline for each simulation condition. We contrast this with findings from a relatively narrow, but non-Rasch discrimination range and a relatively wide one. These ranges are again based on our experience working with educational test data, as well as Domingue (2012b)’s observation that a range of $unif(0.5, 1.5)$ is commonly observed when IRT models are estimated using educational test data. By splitting our non-Rasch conditions into two, we can better assess the extent to which mild or severe deviations from Rasch unit discrimination lead to violations of double cancellation.

In all cases, $\theta \sim N(\text{0,1})$. The first round of simulations entails generating 100 datasets from each combination of these design variables. With 24 combinations of the design variables, this produces 2400 total datasets. For each dataset, Domingue (2014)’s test of single and double cancellation is applied to the dataset via the *ConjointChecks* package (Domingue, 2012a) in the statistical programming software R (R Core Team, 2023). Specifically, we use 5000 random three-matrices per dataset, the same approach taken by Domingue (2014), and we focus on the weighted mean proportion of violations as the outcome of interest, based on Domingue (2014)’s finding that it most effectively discriminated between Rasch and non-Rasch data-generating processes. Note that intermittent parallel computing issues prevented a very small number of analyses from completing, but for all conditions, at least 97 iterations ran fully, and the total number of datasets analyzed was 2393.

We report the distribution of weighted mean proportions of violations for each combination of design variables, demonstrating the extent to which Domingue’s approach can differentiate between Rasch-, mildly non-Rasch-, and severely non-Rasch-generated data. These distributions are reported in terms of descriptive statistics (mean and standard deviation) as well as graphically. We then report on the distributional overlap of the weighted means between non-Rasch conditions and the parallel condition with Rasch unit discrimination. We report the standardized mean difference (SMD) with pooled standard deviations (Cohen, 1969) as well as the distribution-free measure of overlap between two sets of values known as the overlapping index, or $\eta$, implemented in the R package *overlapping* (Pastore, 2018; Pastore & Calcagnì, 2019). While the SMD is widely used and familiar to researchers, it also relies on distributional assumptions (i.e., normality) that our results visually do not fulfill, and we report it mainly because of its familiarity and the fact that our graphical results limit the potential risk of over-interpretation. The advantages of $\eta$ are (1) it requires no distributional assumptions about the sets of values being compared, and (2) its interpretation is simply as the proportion of the two empirical distributions that overlap, with a minimum value of 0 and a maximum value of 1.

### Results

#### Summaries of distributions

**Fig. 1:**
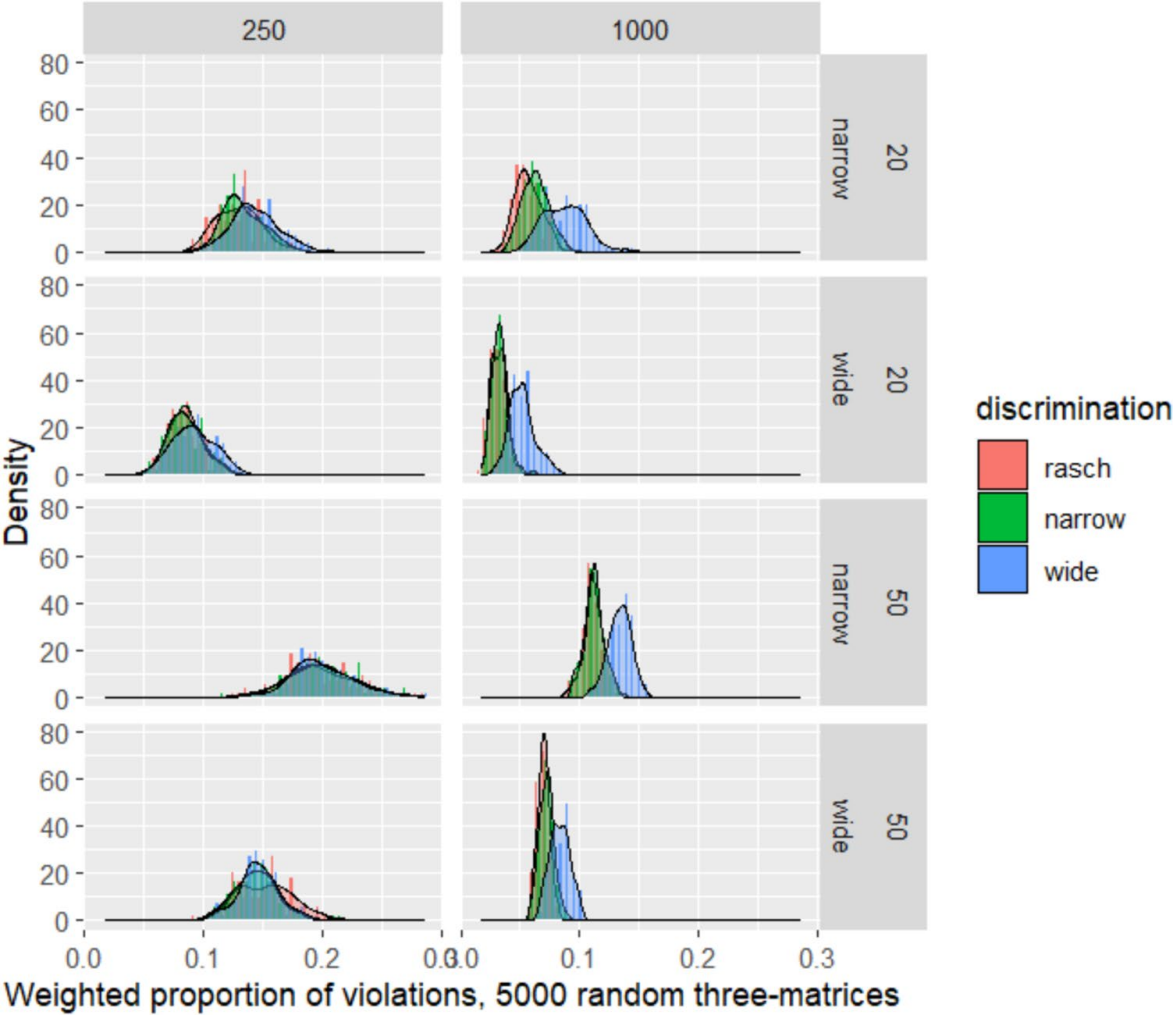
Raw distributions of weighted mean proportions of violations based on BCC procedure

**Fig. 2:**
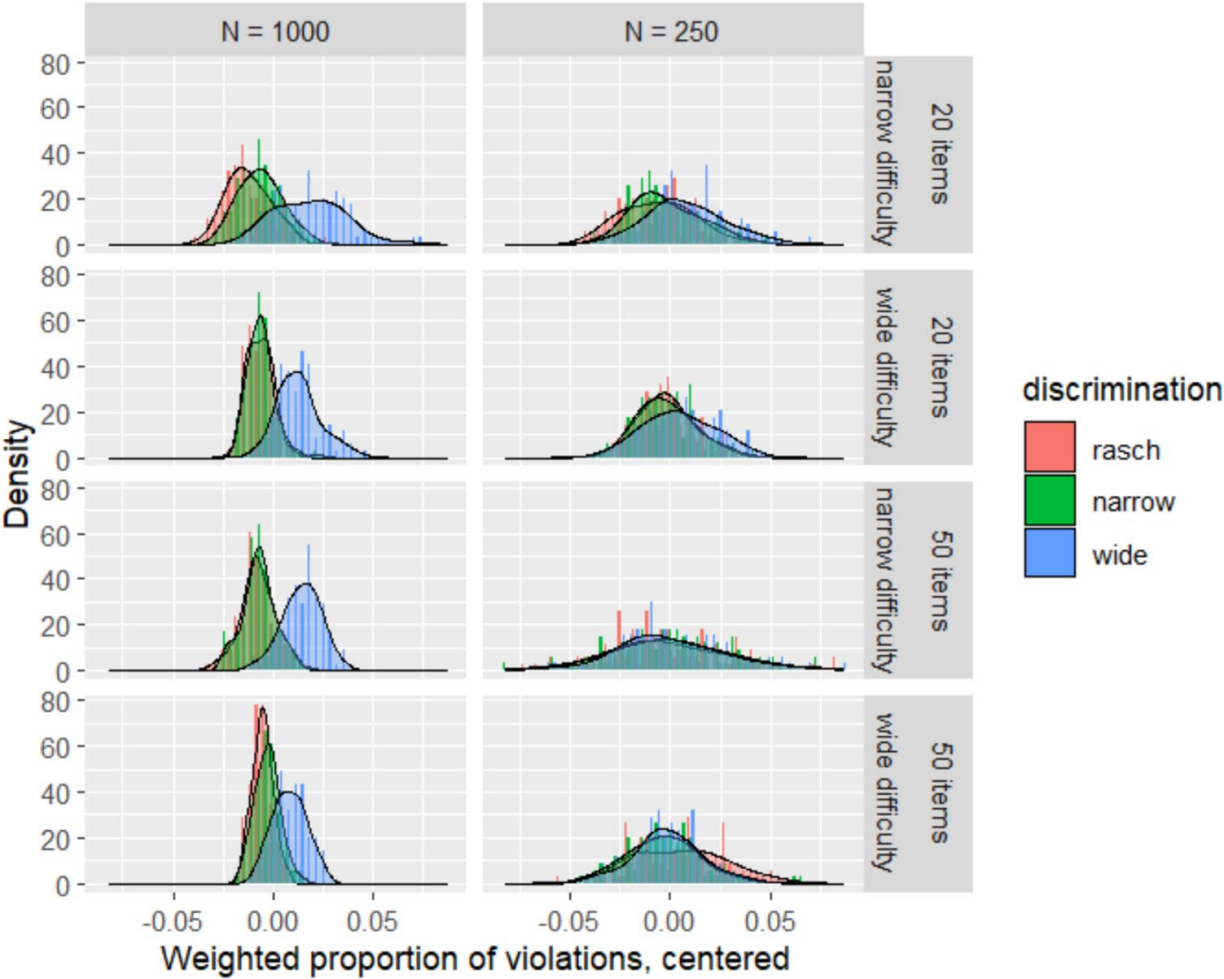
Distributions of weighted mean proportions of violations based on BCC procedure, centered within each cell to highlight contrasts across discrimination conditions

Table 2 presents the distributions of the mean weighted proportion of violations across all replications of each combination of design variables. These distributions are presented graphically in Fig. 1, where the distributions are presented without centering, and Fig. 2, where each cell is centered separately to reduce empty space and focus on comparisons with each cell. Immediately, it should be clear that the behavior of the statistic of interest differs substantially by sample size. At a sample size of 1000, there is clear visual separation between the distribution of values when item discrimination is widely varying, and the distributions when the data-generating discriminations vary narrowly or not at all. In contrast, the narrow discrimination condition is visually only marginally distinguishable from the Rasch unit discrimination condition. This is borne out by the results in Table 2 for *N* = 1000: the means for Rasch and narrow discrimination conditions differ only at the third decimal place, but the means for the wide discrimination condition differ from the other two at the second decimal place.

**Table 2:** Distributions of proportions of violations

Mean (SD) Weighted proportion of violations					
Sample size	Items (#)	Diff. spread	Rasch	Narrow	Wide
1000	20	Narrow	0.057 (0.011)	0.063 (0.011)	0.087 (0.018)
1000	20	Wide	0.032 (0.006)	0.033 (0.007)	0.051 (0.011)
1000	50	Narrow	0.112 (0.009)	0.112 (0.008)	0.134 (0.01)
1000	50	Wide	0.07 (0.005)	0.072 (0.006)	0.083 (0.008)
250	20	Narrow	0.129 (0.018)	0.133 (0.018)	0.143 (0.02)
250	20	Wide	0.085 (0.014)	0.085 (0.015)	0.091 (0.018)
250	50	Narrow	0.199 (0.031)	0.199 (0.029)	0.2 (0.027)
250	50	Wide	0.152 (0.023)	0.145 (0.02)	0.146 (0.017)

By contrast, at *N* = 250, the distributions are barely distinguishable in any systematic manner. Means are quite close to one another across all three discrimination conditions, and with 50 items of widely varying difficulty, the mean value for Rasch conditions is in fact slightly higher than for the other two conditions. We also note that the proportion of violations for each combination of test length/difficulty distribution/discrimination distribution is consistently higher when *N* = 250 compared to *N* = 1000.

#### Distributional overlap

Table 3 presents the overlap between the distribution of values from each non-Rasch condition and its corresponding Rasch condition. Again, at *N* = 1000, there are clear differences between narrow and wide discrimination conditions: uniformly, the proportion of overlap, $\eta$, is more than twice as high for narrow discrimination as it is for wide discrimination, and SMDs are correspondingly much higher for wide discrimination conditions. It is also worth noting that SMDs for the narrow condition are consistently positive and, when the difficulty spread is wide, is 0.4–0.5 SD higher than for Rasch data, even though these conditions’ means only differ at the third decimal place (or smaller) in Table 1. This underscores that even very small differences in the weighted mean proportion of violations may be meaningful for distinguishing interval scalable from non-interval scalable data.

**Table 3:** Measures of distributional overlap between non-Rasch and Rasch weighted proportions of violations, by condition

Sample size	Items (#)	Diff. spread	Discrimination	SMD	$\eta$
1000	20	Narrow	Narrow	0.528	0.785
1000	20	Narrow	Wide	2.042	0.345
1000	20	Wide	Narrow	0.097	0.91
1000	20	Wide	Wide	2.211	0.249
1000	50	Narrow	Narrow	0.056	0.93
1000	50	Narrow	Wide	2.363	0.262
1000	50	Wide	Narrow	0.415	0.817
1000	50	Wide	Wide	1.971	0.347
250	20	Narrow	Narrow	0.234	0.875
250	20	Narrow	Wide	0.733	0.755
250	20	Wide	Narrow	– 0.005	0.958
250	20	Wide	Wide	0.397	0.821
250	50	Narrow	Narrow	0.004	0.941
250	50	Narrow	Wide	0.038	0.919
250	50	Wide	Narrow	– 0.339	0.805
250	50	Wide	Wide	– 0.266	0.795

*Note.* SMD = standardized mean difference with pooled standard deviation. $\eta$ = distribution-free overlapping index (Pastore & Calcagnì, 2019)

However, at *N* = 250, distinctions are far less sharp, in line with the findings above. Here, distinctions between narrow and wide discrimination conditions are only really distinguishable for the shorter test condition of 20 items. For longer tests, there is essentially no way to know if a given weighted mean proportion on its own is indicative of interval scalable data or not. Underscoring this, we note that SMDs for *N* = 250 with a wide spread of item difficulty are negative, meaning that non-Rasch discrimination conditions produced lower proportions of violations.

#### Summary

We take these findings to indicate that extra care must be taken to apply BCC at moderate sample sizes, and that prospects at very small sample sizes (*N* = 250 here) are dim. While there are clear differences between distributions of values at *N* = 1000, even there, there is a fair amount of overlap in the distributions (e.g., even the conditions with the lowest $\eta$ still have ~25% overlap). At *N* = 250, distributions do not appear to differ substantially based on item discrimination distribution differences. Still, recall that these results are based on random draws of parameters from probability distributions that differ by the range of item discriminations. This suggests that, unlike at very large samples (Domingue, 2014), simulating data from approximately similar sets of item parameters and comparing the resulting proportion of violations to the proportion from a real data analysis is not likely to detect violations consistently. We also found evidence of mean violation rates in excess of 0.05 at both sample sizes when data are generated from the Rasch model, further substantiating our claim that the 0.05 cutoff is not generally applicable and the cutoff should be tailored to one’s dataset. Given this, we turn to Study 2 to investigate the extent to which bootstrapping might be used to generate useful null distributions at the sample sizes under investigation.

## Making BCC results interpretable with bootstrapping: Methods, simulation design, and results

Given the findings of simulation Study 1, we now develop a bootstrapping approach to aid in the interpretation of the weighted mean proportion of violations produced by BCC. Below, we outline the proposed method; describe a simulation study to evaluate its performance; and provide the results of this study.

### Use of simulation to investigate interval scaling

In the real data analysis portion of his paper, Domingue (2014) compared the proportion of violations of single and double cancellation in a real dataset to the proportion from generating data using the Rasch model with a similar, but not identical, sample size and test length. In very large samples, the proportion of violations of ACM is likely quite stable across replications and small changes to the number of examinees/items, so if one finds that the proportion of violations in real data is, e.g., more than twice as high as the proportion resulting from Rasch data generation of similarly sized data (as Domingue found in the analysis of reading exam data), this can likely be taken as evidence of the data not adhering to ACM. However, as samples get smaller, this approach may become less defensible. This is because sampling variability becomes more of an issue as samples get smaller, and even relatively minor differences in test length or examinee sample size could lead to meaningfully different proportions of violations. To that end, we suggest two procedures as potential supplements to BCC that can support the productive application of the approach. These procedures are the subject of our second simulation study.

Consider a scenario in which a researcher has run the BCC procedure via *ConjointChecks* on real data and found a proportion of violations greater than 0 (this is essentially inevitable, even with data simulated from the Rasch model). The researcher is unsure if the value indicates incompatibility with ACM. We suggest the following (and investigate its performance via simulation below). First, the researcher can bootstrap a null distribution for the proportion of violations expected when data *are* compatible with an interval scale. The researcher will do so by fitting the Rasch model to their data to produce item and person parameter estimates. For a large number (e.g., 100) of replications, the response probabilities derived from estimated person and item parameters are then used to generate a new item response dataset via weighted draws from the binomial distribution. Each of the datasets is analyzed using *ConjointChecks*, and the resulting distribution of weighted mean proportion violations is then constructed. The empirical violation rate is compared to this distribution, and the data are deemed incompatible with ACM if the empirical violation rate exceeds a predefined threshold (e.g., the 90th, 95th, or 99th percentile of the interval).

Given the findings from Study 1 reported above, we find evidence that the performance of BCC differs substantially across the two sample sizes studied, and that a bootstrapping procedure for the null distribution of violations therefore is also expected to perform differently across the sample sizes. We thus present the results of a second study considering the application of bootstrapped null distributions and filtering of items based on Rasch fit statistics. Given the computational requirements of the procedure described below, this study considers a limited set of data-generating conditions based on Study 1^5^. Previewing our findings, these conditions were chosen to illustrate the contrast in performance across sample sizes. No simulation study is ever comprehensive, and we do not present this study to make a general claim that the procedures we develop *always* work at one sample size and *never* work at the other, but this study shows that sample size does appear to drive major differences in performance in the context of our data-generating models, and lays out methods that researchers can use to decide if our methods are appropriate for analyzing their data by running a similar study to ours with data-generating parameters based on their own data.

This study focuses on defining a procedure for making the weighted mean proportion of violations estimated by *ConjointChecks* more interpretable. The procedure we define, applied to a given item response dataset for which a weighted mean proportion of violations has already been computed, is:

1. Fit the Rasch model to the dataset using conditional maximum likelihood item parameter estimation and maximum likelihood person parameter estimation with spline interpolation for 0/perfect scores via the *eRm* R package (Mair and Hatzinger, 2007);
2. Generate 100 item response datasets by simulating item responses using the Rasch model with parameters for examinee ability and item difficulty from (1);
3. Compute the weighted mean proportion of violations for each generated dataset;
4. Compare the actual proportion of violations to the null distribution of violations defined by the results of (3), with the percentile treated similarly to a *p* value.

The result of this procedure is information about the extent to which the weighted mean proportion of violations that the researcher observed is likely to have arisen via sampling variability alone if the true item response generating process followed the Rasch model. This is operationalized as the percentile of the observed weighed mean proportion of violations within the distribution of weighted mean proportions resulting from simulation of item responses based on the parameters of a Rasch model fitted to the data. If that percentile falls above a certain cutoff – say, the 95th percentile – then the hypothesis that the data are compatible with interval scaling is rejected.

#### Simulation Study 2 design

This study considers a limited set of salient conditions from Study 1. As laid out below, Study 1 indicates that BCC can discriminate much more effectively at larger sample sizes (*N* = 1000 vs. 250) between (1) Rasch-generated item responses vs. (2) severely non-Rasch item responses for which the hypothesis of interval scaling should be rejected. Given this, we investigate how the procedure laid out in the previous section works for 50 items with wide discriminations (i.e., severely non-Rasch) and wide item difficulties at *N* = 250 and *N* = 1000. The first condition is close to a worst-case scenario, based on Study 1. If BCC cannot distinguish between Rasch and severely non-Rasch data at *N* = 250, it would be surprising to find that null distributions and filtering on item fit reliably improve scales at this sample size. Essentially, we investigate this procedure at *N* = 250 under the hypothesis that the procedure *will not work*, and that researchers should be aware of this limitation. On the other hand, at *N* = 1000, BCC appears to distinguish quite well between interval-scalable and non-interval-scalable data, based on Study 1. Here, we expect to find that the proposed method performs more reliably. Importantly, this is a new procedure, and researchers interested in using it are advised to replicate this simulation study with data-generating parameters tailored to their own data, rather than basing their decisions on our results here alone. For comparison, we also run these simulations with Rasch unit discrimination instead of varying discriminations, providing evidence on how the procedure works when the data truly are generated from interval-scaled item and person parameters.

For each sample size and discrimination condition, for 100 replications, we do the following:

1. Generate an item response dataset with 50 items, difficulty drawn from the $N(0, 1.44)$ distribution, and discrimination either drawn from the $U(0.5, 1.7)$ distribution (non-Rasch condition) or assigned a value of 1 (Rasch condition).
2. Use BCC to get the weighted mean proportion of violations for this dataset.
3. Generate a null distribution for this weighted mean proportion of violations, and locate the estimate in this null distribution.
4. Save all of the results, and the data-generating item parameters, for further analysis.

#### Simulation Study 2 evaluation

Evaluating this study is a matter of quantifying both (1) the extent to which the procedure flags non-interval data reliably, (2) how the procedure handles data actually generated by the Rasch model. First, we report, via descriptive statistics, the distribution of the weighted mean proportion of violations under each condition. This tells us the extent to which the outcome statistic itself appears to vary by sample size and true item discrimination. This is already known from Study 1, but leads into the following results: we situate each weighted mean proportion of violations in its bootstrapped null distribution. Here, we again descriptively summarize the percentile of each value in its null distribution. For non-Rasch conditions, we are answering the question: does the procedure flag data not generated from the Rasch model as such? For Rasch conditions, we are answering the question: does the procedure flag data generated from the Rasch model when it should not?

### Results

Below, we review how well our proposed bootstrapping procedure to generate a null distribution works at flagging non-interval data.

#### Outcome descriptive statistics

First, we report the distribution of the weighted mean proportion of violations for each of the four conditions simulated in Table 4. From these results, it is apparent that the weighted mean proportion of violations is consistently much higher at *N* = 250, but only differentiated between Rasch and non-Rasch conditions at *N* = 1000, in line with Study 1. However, the main result of interest is the extent to which the generation of a null distribution can accurately contextualize these outcomes – the hope is that the weighted mean proportion of violations will tend to fall near the middle of its null distribution with item response data generated from the Rasch model, but at the upper edge of the distribution when the data are generated with varying item discriminations.

**Table 4:** Distribution of weighted mean proportion of violations

Sample size	*N* = 250		*N* = 1000	
Discrimination	Rasch	Non-Rasch	Rasch	Non-Rasch
N Replication	100		100	
Mean	0.149	0.148	0.070	0.084
Median	0.149	0.146	0.069	0.084
SD	0.020	0.021	0.005	0.007
Min.	0.107	0.090	0.059	0.066
Max.	0.216	0.200	0.083	0.103
5th percentile	0.123	0.112	0.062	0.072
95th percentile	0.181	0.184	0.079	0.095

#### Assessment of interval scaling

Recall that the result of the procedure under investigation is a percentile of the weighted mean proportion of violations from BCC within its simulation-based null distribution. A value close to 1 indicates that the observed value was unlikely to have arisen by chance if all items had unit discrimination, so the dataset is rejected as incompatible with interval scaling if this value is above a set cutoff (e.g., the 90th, 95th, 99th percentile). Table 5 reports descriptive statistics for this outcome at *N* = 250 and *N* = 1000. We report the mean, median, standard deviation, and minimum of the percentile of the proportion of violations from BCC relative to its null distribution across the 100 replications. We also report rates at which interval scaling is rejected at different cutoff values. There are very sharp contrasts between results at *N* = 250 and *N* = 1000. At *N* = 250, the procedure essentially does not work at all. Data generated from a non-Rasch set of discrimination parameters are rarely flagged for lack of compatibility with interval scaling. At N = 1000, on the other hand, results are quite consistent, with 97% of runs rejected at a cutoff of the 95th percentile of the null distribution. Results from conditions Rasch-generated show the same contrast: at *N* = 250, results generated from Rasch and non-Rasch models look nearly identical, while they are quite differentiated at *N* = 1000.

**Table 5:** Results of initial comparison of simulated datasets to their null distributions

Sample size	*N* = 250		*N* = 1000	
Data generation	Rasch	Non-Rasch	Rasch	Non-Rasch
*N* Replication	100	100	100	100
Mean percentile	55.4	62.2	44.6	99.21
Median percentile	57.5	67	42.0	100.0
SD percentile	24.6	24.9	29.4	3.8
Min. percentile	3.0	4.1	0.0	67.0
Prop. rejected, 90% cutoff	0.10	0.13	0.05	0.98
Prop. rejected, 95% cutoff	0.02	0.06	0.03	0.97
Prop. rejected, 99% cutoff	0.01	0.01	0.01	0.87

*Note.* Percentile = percentile of weighted mean proportion of violations for the dataset within its bootstrapped null distribution. Prop. rejected,* x*% cutoff = proportion of runs in which the simulated dataset fell above the *x*th percentile of its null distribution and was therefore flagged for non-interval scaling

### Real data analysis

Given the above results, it appears that BCC with a bootstrapped null distribution for the weighted mean proportion of violations is sensitive to violations of interval scaling with reasonable item parameter distributions and *N* = 1000. We therefore proceed to a real data analysis in which we evaluate an item response dataset with *N* = 1000 for interval scalability.

### Data and methods

We use BCC and a bootstrapped null distribution to analyze a dataset consisting of 1000 students’ responses to 24 math calculation items on the Swedish Scholastic Aptitude Test (SweSAT) (Stage & Ögren, 2004). This dataset is available in the R package *TestGardener* (Li et al., 2019), and we obtained it via the Item Response Warehouse, a public item response data repository (Domingue et al., 2023). This section of the SweSAT is an example of the sort of educational or psychological test that is often used to produce (sub)scores for use in correlation-, regression-, or difference-based analyses (e.g., Cliffordson, 2004; Löwenadler, 2023), but was not, to our knowledge, explicitly created to produce an interval scale. Our procedure to analyze the dataset involves both establishing that the analysis is powered to detect violations, and then analyzing the actual dataset. The full procedure is as follows:

1. Fit the Rasch model to the data using conditional maximum likelihood item parameter estimation and maximum likelihood person parameter estimation with spline interpolation for 0/perfect scores via the *eRm* R package  (Mair and Hatzinger, 2007);
2. Confirm that there is sufficient sensitivity to detect violations of interval scaling in data with this sample size and distribution of item/person parameters using simulation;
3. Compute the weighted mean proportion of violations in the dataset using *ConjointChecks*;
4. Generate 100 item response datasets by simulating item responses using the Rasch model with parameters for examinee ability and item difficulty from (1);
5. Compute the weighted mean proportion of violations for each generated dataset;
6. Compare the actual proportion of violations to the null distribution of violations defined by the results of (5), treating the 95^th^ percentile as out cutoff for establishing a violation of interval scalability.

We begin by fitting the Rasch model, per step 1, and estimating person parameters. We then conduct a simulation study similar to Study 2 to verify that the procedure is sensitive to deviations from unit discrimination/interval scaling. While the sample size is 1000, this dataset includes 24 items, rather than the 50 in Study 2. We re-run the first stage of Study 2 (the initial assessment of interval scaling) with *N* = 1000, 24 items, item difficulty distribution $b\sim N(-0.04, 0.34)$, $\theta$ distribution $\theta \sim N(-0.18, 0.90)$ and discrimination distribution matching the prior study at $a\sim U(0.5, 1.7)$. These item difficulty and $\theta$ distributions are based on the item and person parameters produced when fitting the Rasch model to real data, so if the simulations indicate reasonable sensitivity under these data-generating conditions, then we can be confident in the results of the real data analysis. That is, in fact, what we find. As reported in Table 6, 98% of simulated datasets are flagged as not compatible with interval scaling when the proportion of violations computed for the data is compared to the 95th percentile of the relevant bootstrapped null distribution of proportions of violations, so sensitivity is sufficient. From here, we proceed to the main analysis of the data. We compute the proportion of violations in the dataset and compare it to the bootstrapped null distribution based on the estimated parameters for the dataset^6^.

**Table 6:** Results of comparing simulated datasets mimicking SweSAT data to their null distributions of proportions of violations

Sample size	*N* = 1000
N Replication	100
Mean percentile	99.7
Median percentile	100.0
SD percentile	1.8
Min. percentile	83.0
Prop. rejected, 90% cutoff	0.99
Prop. rejected, 95% cutoff	0.98
Prop. rejected, 99% cutoff	0.91

*Note.* percentile = percentile of weighted mean proportion of violations for the dataset within its bootstrapped null distribution. Prop. rejected,* x*% cutoff = proportion of runs in which the simulated dataset fell above the *x*th percentile of its null distribution and was therefore flagged for non-interval scaling

### Result

**Fig. 3:**
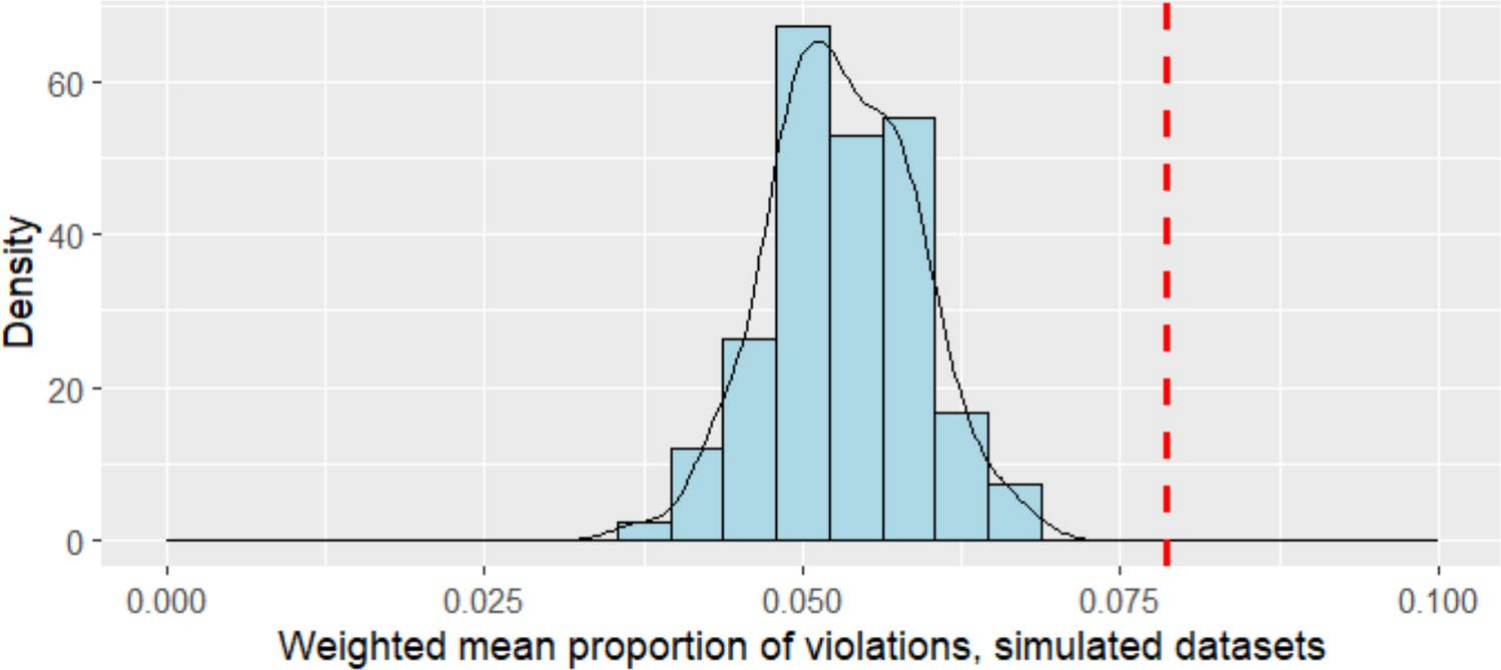
Empirical value (*red*) and null distribution (*blue*) of weighted mean proportion of violations for SweSAT data

The item response data that we analyze are quite clearly incompatible with the construction of an interval scale using the Rasch model: Figure 3 plots the null distribution compared to the empirical proportion of violations. The weighted mean proportion of violations of single and double cancellation in the dataset is 0.081, while the maximum value of this statistic in the null distribution based on simulation from the Rasch model is 0.069 (mean = 0.054, SD = 0.0060). That is, the empirical weighted mean proportion is higher than any value in the null distribution (100th percentile). As such, we would reject the claim that the Rasch model, fit to these data, produces an interval scale. This is the case even though the data appear to fit well using mean square fit statistics widely used in Rasch analysis (Linacre, 2002; Rasch, 1960; Wright & Linacre, 1994; Wright & Stone, 1979): infit and outfit, respectively, range from 0.85 to 1.20 (mean = 1.00, SD = 0.11) and from 0.81 to 1.24 (mean = 1.00, SD = 0.11). This is similar to a result originally observed by Karabatsos (2001): “rule of thumb” Rasch fit statistics and their traditional ranges (e.g., 0.7–1.3) should not be relied upon to substantiate a claim that a scale based on a Rasch calibration possesses interval properties.

### Discussion

This study investigated how the BCC method for assessing adherence of item response data to additive conjoint measurement axioms performs and can be applied to real data, at moderate sample sizes. Our first simulation study provided evidence that the results of BCC vary considerably by sample size under our data-generating item and person parameter distributions. The second simulation study evaluated the use of a null distribution based on estimated Rasch model parameters to contextualize the results of applying BCC to real data. Finally, we presented a real data analysis demonstrating the use of a simulated null distribution to reject the hypothesis that a given item response dataset for 1000 examinees is compatible with interval scaling. We concluded that the BCC procedure itself appears to require a larger sample than 250 when the data-generating item and person parameters follow the distributions we specified. Note that this does not mean that the procedure cannot *ever* work at *N* = 250; no simulation study, especially one with a limited number of conditions such as ours, is comprehensive. Still, if one accepts the idea that discriminations between 0.5 and 1.7 are widely distributed enough to constitute violations of unit discrimination, which we think should be uncontroversial, then it appears that prospects for using BCC at *N* = 250 are poor. On the other hand, the procedures we develop in this manuscript appear to discriminate interval scalable from non-interval scalable data quite well at *N* = 1000, providing evidence that investigating the interval properties of scales at this sample size is indeed possible. Of course, future simulation studies with similar sample sizes but very different data-generating parameters might produce different findings, underscoring the fact that – as we enact in the real data analysis – simulations similar to ours should always be used to verify that the procedure is sensitive to departures from Rasch-generated data at the sample size, test length, and parameter distributions found in one’s actual data before applying the procedure. It is important to recognize that varying item discrimination is not the only phenomenon that can drive violations of the cancellation axioms; guessing behavior can do this as well, as investigated by Domingue (2014), and we speculate that other empirical departures from the Rasch model found in the study of academic growth such as empirically asymmetric item response functions (Bolt et al., 2014; Liao & Bolt, 2021; Samejima, 2000) or differential item functioning (Student, 2025) would also lead to violations. We hope that researchers will take up the general framework we present in this study to investigate the interval properties of more educational and psychological scales, and to gain a better understanding of how different item response behaviors and patterns drive violations of the cancellation axioms.

Another future direction is to investigate the speculation that mean-square fit statistics might act as quantifications of item-level departures from an interval scale (Karabatsos, 2001). Karabatsos notes that these statistics are sensitive to empirical departures from the Rasch model’s unit discrimination specification, though more recent research has indicated that these statistics do not always function well in practice (Müller, 2020; Wu & Adams, 2013), even if they are commonplace in Rasch analysis (T. G. Bond & Fox, 2015; Wright & Linacre, 1994; Wright & Stone, 1979). Other item fit approaches, including item-specific violation rates from *ConjointChecks* or measurement error-corrected item fit statistics (Chalmers & Ng, 2017), might perform better than infit and outfit for the task of selecting interval-scalable items from a larger set.

We also note as a future direction the prospect of error correction in the bootstrapping procedure more broadly. We used estimated item and person parameters, which inevitably contain random measurement error that inflates the variance of both distributions. This may be particularly relevant for the person distribution, as $\theta$ estimates tend to have larger standard errors than item parameter estimates. We speculate that the performance of the bootstrapping method might be improved in smaller samples by applying a small amount of shrinkage to the parameter estimates used for bootstrapping to counteract their slightly inflated variance. Given the contrast in our findings between *N* = 250 and *N* = 1000, these corrections are likely especially relevant at sample sizes above 250 (where the investigated procedure was not successful) and below 1000 (where it was). Future work should consider how different approaches to correcting for this error might further improve the performance of the methods we developed in this manuscript, while also recognizing that Rasch analysis, especially conditional maximum likelihood estimation, intentionally avoids placing a parametric distribution on person or item parameters.

Expansion to other measurement models is another clear area for innovation. Rasch-type polytomous item response models (Andrich, 1978; Masters, 1982) seem like a natural area for further development, especially because many educational tests consist of a mixture of dichotomous and polytomous items. Extension to non-Rasch IRT models using polynomial conjoint measurement (Krantz et al., 1971; Tversky, 1967) would also represent a major contribution in this area. These areas are both much more complex than the dichotomous Rasch case considered in this paper.

We end by noting that it is one thing to identify a violation of double cancellation, but another to understand the practical significance of a violation. It strikes us as plausible that many analyses of item and/or person parameter estimates (i.e., scores) will be relatively robust to small violations of the cancellation axioms, but there is evidence that certain types of analysis such as regression with interactions (Domingue et al., 2022) and analysis of growth over time (Briggs, 2013) can be quite sensitive to non-interval scales. In some cases, it may be defensible to just remove a subset of items from the scale in the name of improving its interval properties – specifically, if removing those items does not affect representation of the underlying construct that the researcher intends to measure (see, e.g., Wilson, 2023). That said, if the items are pervasively incompatible with an interval interpretation, or if they are all critical to content representation, this route may not be viable. We thus emphasize the importance of research that aims to quantify the implications of non-interval scaling for common data analyses. This will inevitably take a variety of forms depending on the aims of a given analysis. One possible route for future methodological development is sensitivity/robustness analysis focused on inducing non-interval scaling in simulated data, and assessing the ways that common analyses are or are not robust to these manipulations (e.g., Briggs & Domingue, 2013; Domingue et al., 2022; Liao et al., 2024; Zumbo & Zimmerman, 1993). This type of analysis can help applied researchers understand how far off the answers to their substantive questions may be if they treat non-interval data as interval. Another possible route that may be viable for some intended uses of test/survey scores, particularly direct inferences about individuals’ proficiencies, attitudes, etc., is to adopt methods that are not premised on interval scales or quantitative structure in the first place (Scharaschkin, 2024). We hope that researchers will continue to grapple with the metric properties of psychological scales on multiple fronts to build the field’s understanding of when these issues matter most.

## Footnotes

1 For a useful discussion of the term “meaningfulness” in this context, see Michell (1990, p. 44).

2 I.e., (1) the “Archimedean” condition that no value of any variable is infinitely larger than the value of any other variable, and (2) the “solvability” condition that for any combination of independent variables, there is another combination of independent variables that produce the same value of the dependent variable.

3 Note that for all but the shortest tests, evaluating all possible 3-matrices is computationally burdensome in the extreme; as Domingue notes, a 45-item test with a reasonable spread of sum scores would have over ${10}^{8}$ possible 3-matrices.

4 The vigorous debate on this issue and the prospect of quantitative measurement in psychology (examples include Borsboom & Mellenbergh, 2004; Humphry, 2013; Krantz & Wallsten, 2019; Michell, 2000, 2008, 2014, 2019; Sijtsma, 2012; Trendler, 2009, 2018, 2022) is not considered in depth in this manuscript.

5 On a research computer with 24 cores, the simulations for Study 2 take approximately 1 week to run.

6 All code used in this analysis, and instructions for downloading the data, are available via an Open Science Foundation repository, https://osf.io/8vqg5/?view_only=a2f8ef2aff934009924ccd084f173798.
